# A novel SYBR-based duplex qPCR for the detection of gene dosage: detection of an *APC* large deletion in a familial adenomatous polyposis patient with an unusual phenotype

**DOI:** 10.1186/1471-2350-13-55

**Published:** 2012-07-16

**Authors:** Giovana Tardin Torrezan, Felipe Cavalcanti Carneiro da Silva, Ana Cristina Victorino Krepischi, Érika Maria Monteiro dos Santos, Benedito Mauro Rossi, Dirce Maria Carraro

**Affiliations:** 1 CIPE - International Center of Research and Training - A. C. Camargo Hospital, Rua Taguá, 440, CEP: 01508-010, São Paulo, SP, Brazil; 2 National Institute of Science and Technology in Oncogenomics (INCITO), São Paulo, SP, Brazil; 3 Barretos Cancer Hospital - Pio XII Foundation, Barretos, SP, Brazil

**Keywords:** Gene dosage, Quantitative PCR, Familial adenomatous polyposis, *APC* whole gene deletion

## Abstract

**Background:**

Familial adenomatous polyposis (FAP) is a hereditary colorectal cancer syndrome caused by a loss of function of the *APC* gene. Large deletions in *APC* are a common cause of FAP; despite the existence of a variety of gene dosage detection methodologies, most are labor intensive and time and resource consuming.

**Methods:**

We describe a new duplex qPCR method for gene dosage analysis based on the coamplification of a target and a reference gene in a SYBR Green reaction, followed by a comparison of the ratio between the target and the reference peaks of the melting curve for the test (patient) and control samples. The reliability of the described duplex qPCR was validated for several genes (*APC*, *HPRT1*, *ATM*, *PTEN* and *BRCA1*).

**Results:**

Using this novel gene dosage method, we have identified an *APC* gene deletion in a FAP patient undergoing genetic testing. Comparative genomic hybridization based on microarrays (aCGH) was used to confirm and map the extent of the deletion, revealing a 5.2 MB rearrangement (5q21.3-q22.3) encompassing the entire *APC* and 19 additional genes.

**Conclusion:**

The novel assay accurately detected losses and gains of one copy of the target sequences, representing a reliable and flexible alternative to other gene dosage techniques. In addition, we described a FAP patient harboring a gross deletion at 5q21.3-q22.3 with an unusual phenotype of the absence of mental impairment and dysmorphic features.

## Background

Familial adenomatous polyposis (FAP) is a dominantly inherited syndrome characterized by the development of hundreds of adenomatous colorectal polyps and, consequently, colorectal cancer (CRC) [1,2]. FAP is caused by mutations in the tumor suppressor gene *APC* (adenomatous polyposis coli - NM_000038.5) mapped at 5q21-q22 [3,4]. Over 1100 different pathogenic *APC* mutations have been reported to date (LOVD Mutation Database - http://www.lovd.nl/2.0/), and the great majority are nonsense mutations or small deletions and insertions leading to a truncated protein. Large gene deletions account for approximately 5% of the germline *APC* mutations, but the true prevalence of this type of alteration remains unknown due to the lack of easy screening techniques [5]. Such alterations have been detected by Southern blotting, MLPA®, competitive and differential qPCR, long-range PCR and DNA array-based methods [6]. Despite the great variety of techniques that exist to determine gene dosage, most of them are labor intensive and time and resource consuming.

The aim of this study was to develop a novel gene dosage method using SYBR Green to confirm a genomic alteration in a FAP patient. The technique is based on the coamplification, by duplex PCR, of the target and reference genes and the comparison of the peaks of the melting curve (PMc) ratio between patient and controls. This method was successfully validated and applied to identify a large genomic deletion encompassing the *APC* and 19 additional genes. Thus, this paper presents the detailed description of this novel gene dosage method, as well as evaluates the genotype-phenotype correlation of the *APC*-deleted patient.

## Methods

### Samples

The index patient (FAP02) was a Brazilian male with clinically suspected FAP identified in the Hereditary Colorectal Cancer Registry of AC Camargo Hospital (São Paulo, Brazil). His family history was accessed through the index patient report: his paternal grandmother, his father and one paternal uncle were affected with polyps and CRC at unknown ages, and one sister presented with polyps/CRC at the age of 44 that progressed to liver metastasis. The affected relatives were deceased; therefore, no biological material was available for mutation screening. One unaffected sister and one unaffected niece were available for genetic testing (Additional file 1: Figure S1). The index patient was diagnosed with colorectal cancer (T1N0M0) at the age of 40, and harbored more than 100 synchronous adenomatous colorectal polyps, including duodenal and gastric polyps; no signs of other extracolonic manifestations commonly associated with FAP were observed. The patient was referred for total proctocolectomy with ileoanal pouch anastomosis. Examinations performed by a gastroenterologist and a genetic counselor did not identify any dysmorphic features or severe mental impairment. The patient completed his high school education at a regular school, and then has been working and living independently.

The validation of the gene dosage assay was performed on samples from 24 healthy controls and three patients with previously detected genomic alterations. In addition, the novel missense variant identified in the FAP patient was screened for in 95 healthy individuals.

This study was executed in compliance with the Helsinki Declaration and was approved by the ethics committee of AC Camargo Hospital (approval number: 1169/08-B). Written informed consent was obtained from all patients and controls. Genomic DNA from patients and controls was obtained from blood samples using the Puregene Genomic DNA Isolation Kit (Gentra Systems - Minneapolis, MN, USA) according to the manufacturer’s instructions. The DNA concentration was verified with a NanoDrop 1000 Spectrophotometer (Thermo Fisher Scientific – Waltham, MA, USA) and DNA samples were diluted to a concentration of 25 ng/μL. Immediately before the qPCR assay, the concentration of DNA samples was reassessed and they were diluted to a 3.6 ng/μL solution, from which 5 μL was used in each qPCR reaction (a total of 18 ng).

### PCR and sequence analysis

Sanger sequencing of all *APC* exons (NM_000038.5), including intron-exon boundaries, was performed in the FAP patient. The amplified fragments were directly sequenced in both directions using the ABI3130xl sequencer (Applied Biosystems - Foster City, USA). The output results were aligned to the reference sequence using CLC Bio Main Workbench Software (Muehltal, Germany). In addition, the presence of the novel missense variant was screened for in 95 control samples by PCR followed by sequencing. The primer sequences and PCR conditions are available upon request.

### SYBR-based duplex qPCR

Two main principles were followed for designing the duplex qPCR primers, as follows.

1) The total length of the amplicon (including the primers) should be between 90 and 220 bases.

2) Amplicons for the target and reference genes should have a minimum of 5°C of difference between their melting temperatures (Tm), such that a clear individualization of target and reference gene melting peaks can be achieved.

In all duplex qPCR reactions, the target and reference genes were coamplified with 0.15 μM of each primer, 18 ng of DNA and 1X SYBR® Green PCR MasterMix (Applied Biosystems - Foster City, CA, USA) in a 20 μL reaction. qPCR was performed using an ABI Prism 7500 detection system (Applied Biosystems - Foster City, CA, USA). The amplification conditions were as follows: a 10 min preincubation at 95°C followed by 25 cycles of 15 sec at 95°C and one minute at 60°C. The reduced number of cycles (25) maintains the reaction in the exponential phase, ensuring that the final amount of amplified product (demonstrated by the PMc height) is proportional to the starting amount of template DNA.

After coamplification, the PCR products were subjected to a linear temperature transition from 60°C to 95°C at 0.1°C/s, and melting curves of the decrease of SYBR Green fluorescence were generated using the ABI Prism 7500 software (Applied Biosystems - Foster City, USA). The PMc were identified by plotting the negative first derivate of the change in fluorescence (–dF/dT, the rate of change of fluorescence) vs. temperature, allowing discrimination of the two PMcs, one from the reference gene and the other from the target gene.

The ratio between the target and reference PMc height reflects the relative concentration of the target gene in the sample. Normalizing the PMc target/reference ratio of the patient sample with the PMc ratio of the control sample generates a value that corresponds to the DNA dosage, revealing whether the target sequence is deleted, duplicated or normal. Ratios between 0.9 and 1.1 were considered normal, ratios between 0.6 and 0.7 indicated deletions of one allele, and ratios above 1.4 indicated amplifications; ratios outside these intervals were considered inconclusive and repeated. Values were determined empirically based on the X chromosome dosage assay, target genes validation tests and sensibility assay described below. All the assays included three healthy controls and were performed in duplicate for patients and controls.

Validation of this novel gene dosage assay was accomplished through the assessment of X chromosome dosage in 24 healthy controls (12 males and 12 females) by determining the ratio between *HPRT1* exon 3 (Xq26.1), as the target gene, and *GAPDH* intron 7 (12p13), as the reference gene. A control sample from a female was used to normalize the ratios. Further validation was performed for different target genes (*ATM* exon 4*, PTEN* exon 2 and *BRCA1* exon 24) in samples from patients harboring germline gene deletions and duplications previously detected by aCGH in other studies of our group.

For the FAP patient, the duplex qPCR was performed for *APC* exons 2 and 15 using *GAPDH* as the reference gene. After confirming the *APC* deletion in this patient, the detection sensitivity of this method was assessed by mixing five different DNA proportions of the *APC*-deleted patient (P) and a healthy control (C). We used 18 ng of DNA in each reaction as follows: 1 P : 0 C (18 ng P : 0 ng C); 3 P : 1 C (13.5 ng P : 4.5 ng C); 1 P : 1 C (9 ng P : 9 ng C); 1 P : 3 C (4.5 ng P : 13.5 ng C); and 0 P : 1 C (0 ng P : 18 ng C). All mixtures were submitted to a duplex qPCR for the *APC* exon 15 as the target gene and *GAPDH* as the reference gene. Experiments were performed in duplicate and data obtained from the sample containing only DNA from the wild-type control was used to normalize the PMc ratios.

### aCGH experiments and data analysis

Comparative genomic hybridization based on microarrays (aCGH) was performed in duplicate for patient FAP02 using an 180 K whole-genome platform (OGT – Oxford, UK). Briefly, the samples were labeled with Cy3- and Cy5-dCTPs by random priming. Purification, hybridization, and washing were performed as recommended by the manufacturer. Data extraction was conducted using the Feature Extraction software (Agilent Technologies - Santa Clara, USA). Genomic Workbench software (Agilent Technologies - Santa Clara, USA) was used to identify the constitutive genomic imbalances using the statistical algorithm ADM-2 with a sensitivity threshold of 6.7 and threshold log2 ratios of 0.4 and 1.1 for duplication and high copy number gains, and -0.4 and -1.1 for deletion and homozygous loss, respectively.

## Results

**Figure 1:**
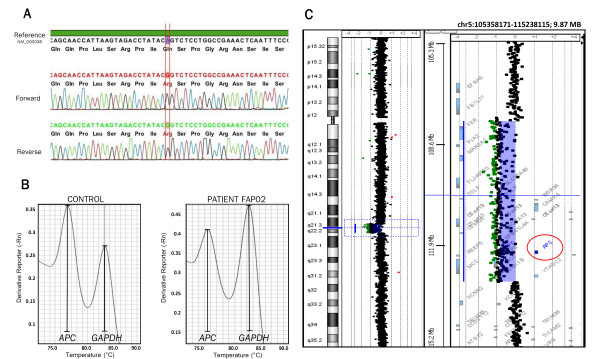
**Missense mutation and deletion of the *APC* gene in one FAP patient. A:** Chromatogram of the novel missense variant c.6965A > G p.Gln2322Arg in *APC* exon 15 apparently in homozygosis. **B:** Melt curve of duplex qPCR of *APC* exon 15 and *GAPDH* intron 7 (reference gene) – PMc ratio of *APC/GAPDH* in the control sample was 1.4 and in the FAP patient it was 0.86, leading to a normalized PMc ratio of 0.61 for the FAP patient, indicating the presence of deletion. **C:** aCGH chromosome 5 profile of the FAP patient showing a 5.2 Mb deletion at 5q21.3-q22.3 that encompasses the entire *APC* sequence (red circle) and 19 additional genes.

A novel homozygous missense variant (c.6965A > G p.Gln2322Arg – Figure 1A) at exon 15 was identified in one FAP Brazilian patient undergoing genetic testing of the *APC* gene (consulted databases: LOVD -http://www.lovd.nl/2.0/, dbSNP -http://www.ncbi.nlm.nih.gov/projects/SNP/, and 1000 Genomes - http://www.1000 genomes.org/)*.* Because FAP is generally characterized by loss of function mutations in only one allele, the presence of an undescribed homozygous missense mutation led us to suspect the existence of a large *APC* deletion in the second allele. To test this hypothesis, we developed and validated a new SYBR® Green-based duplex qPCR method for gene dosage assessment.

**Figure 2:**
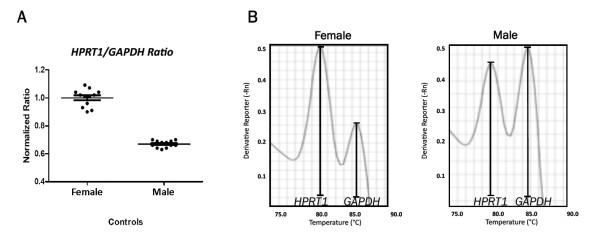
**X chromosome dosage assay for validation of the SYBR green duplex qPCR. A:** Distribution of the normalized *HPRT1* / *GAPDH* ratio in 24 control samples (12 females and 12 males); the normalized ratio of the 12 female samples ranged from 0.90 to 1.09, while in the 12 male samples the range was from 0.63 to 0.70, when using a female sample as the control. **B:** Visual comparison of the melt curve revealing a reduction of the *HPRT1* peak when comparing a male to a female sample.

To determine the ability of our technique to dose chromosomal copy alterations, an X chromosome dosage assessment was carried out. Male and female control samples were evaluated to determine the ratio of the *HPRT1* (Xq26.1) and a reference gene (*GAPDH*). The normalized PMc ratios from the male samples ranged from 0.63 to 0.70, and the ratios from the female samples ranged from 0.90 to 1.09, indicating the presence of one X chromosome in the male samples and two in the female samples (Figure 2A). Visual inspection revealed a clear reduction in the *HPRT1* melting peak in the male samples relative to that in the female samples (Figure 2B).

To further validate this approach for different target genes and samples, we used genomic DNA previously submitted to aCGH in which chromosomal alteration had been detected (data not shown). The duplex qPCR was performed in two samples harboring gene deletions and one sample carrying gene duplication. All genomic alterations were confirmed by this method: the deleted samples showed normalized PMc ratios of 0.63 and 0.64 (*ATM* and *PTEN* genes, respectively), and the *BRCA1* duplicated sample displayed a normalized PMc ratio of 1.5. No overlap between the ratios of any of the wild-type controls and the ratios of the deleted/duplicated patients was detected.

For the *APC* gene, the duplex qPCR was performed for exons 2 and 15, and gave normalized PMc ratios of 0.67 and 0.61, respectively (Figure 1B), revealing a heterozygous deletion for these *APC* exons in the FAP patient. These results were confirmed using standard differential qPCR with 2^-ΔΔ*C*q^ calculations [7] (see Additional file 1, Methods), which revealed values of 0.56 and 0.49 for *APC* exons 2 and 15, respectively. With the purpose of elucidating the extent of the patient *APC* deletion, aCGH was executed using an OGT 180 K whole-genome platform; we detected a 5.2 Mb deletion at 5q21.3-q22.3 – chr5:g.(107,755,923_107,818,559)_(113,079,145_113,113,875)del (UCSC Feb. 2009 – GRCh37/hg19) – that encompasses the entire *APC* gene and 19 additional genes (Figure 1C).

**Figure 3:**
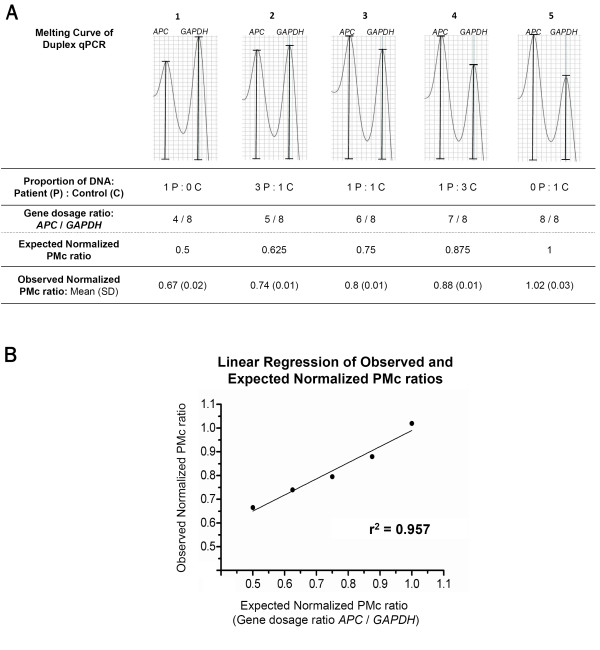
**Sensitivity of the duplex qPCR.** The sensitivity of this methodology was investigated by mixing different DNA proportions of the deleted patient and a healthy control. **A:** First panel presents the melting curve of the five different DNA proportions used (described in the first line of the table); second and third line show the gene dosage ratio between *APC* and *GAPDH* present in each mixed DNA solution and the respective expected normalized PMc ratio; last line presents mean and standard deviation (SD) of the observed normalized PMc ratio duplicates. The results demonstrated that this approach could detect gene dosage alterations even when only 25% of the DNA carried the heterozygous gene deletion (proportion 4 = 1 P : 3 C). **B:** Linear regression of the observed normalized PMc ratio in relation to the expected PMc ratio, demonstrating an r² of 0.957.

Finally, the sensitivity of this method was assessed by mixing different proportions of DNA from the deleted FAP patient and a healthy control (Figure 3A and B). The results demonstrated that this approach could detect gene dosage alterations even when only 25% of the DNA carried the heterozygous gene deletion (proportion 4), making this technology capable of detecting mosaic gene deletions.

## Discussion

We have described a simple and inexpensive duplex qPCR method employing SYBR Green for the assessment of DNA copy number, thereby revealing genomic duplications and deletions. We also identified a FAP patient carrying a 5.2 MB deletion in 5q21.3-q22.3 in addition to a novel missense *APC* variant (p.Gln2322Arg). We examined the presence of this undescribed missense variant in 95 healthy controls, and the alteration was not detected in any control individual. Because FAP is a dominant condition that is usually caused by truncating mutations or large deletions in the *APC* gene, we strongly believe that the causative mutation of the polyposis observed in this family is the large deletion and that the missense variant represents a rare neutral variant in the index patient.

Data regarding clinical characteristics of individuals carrying whole-gene *APC* deletions are limited. However, reported cases have consistently exhibited a degree of polyposis typical of classical FAP (between 100 and 1000 adenomatous polyps) [8-10]. Our patient was diagnosed with colorectal cancer at the age of 40 and harbored more than 100 adenomatous colorectal polyps, including duodenal and gastric polyps, but no other extracolonic manifestations commonly associated with FAP (desmoids, osteomas, cutaneous soft-tissue tumors, dental abnormalities and CHRPE).

Severe to mild mental impairment is an additional common phenotype described for FAP individuals with chromosomal deletions at 5q15-q22, 5q21–q22 and 5q22.1–q31.1, as revised by Readle et al. [9] and described in the Decipher Database (http://decipher.sanger.ac.uk/). Furthermore, those patients have generally been characterized by dysmorphic facial features, including macrognathia, hypertelorism, a high forehead, a well-demarcated philtrum, a high arched palate, and “carp mouth” [10]. The similarities between these patients raise the possibility that the various phenotypes represent a contiguous gene syndrome resulting from partial or complete loss of adjacent genes at 5q, usually occurring as *de novo* mutations, with only a few deletions being described as inherited within families [9]. In these patients, distal deletions (5q22 to 5q31.1) appear to be associated with severe intellectual disability, while deletions encompassing the 5q15 to 5q22 region result in a milder phenotype in terms of both intellectual disability and physical features [8,9]. In our study, the identified gross deletion (5q21.3-q22.3) encompasses the entire *APC* gene and 19 additional genes and is likely to have been present in this family for at least three generations, with a quite unusual phenotype of absence of mental impairment and dysmorphic features. However, mild mental impairment cannot be ruled out because no test was applied to measure the cognitive ability of the index patient.

To date, the most frequently used techniques to detect gene copy number alterations include aCGH, which is expensive; MLPA®, which depends on the availability of the gene of interest; and quantification using real-time PCR, which can be performed with intercalating dyes (such as SYBR® Green) or TaqMan® assays. The latter are more financially onerous because specific primer and probes sets for each target gene must be ordered commercially.

Regular SYBR® Green qPCR can be performed either in a single tube with an internal control fragment (competitive PCR) or using separate reactions for the target and control genes (differential PCR). However, competitive qPCR is a time-consuming process that is limited to sets of primers available from one supplier [11], and the reproducibility of differential qPCR is inevitably compromised by the variable efficiency of the PCR itself because small variations in reaction components can greatly influence the final yield of the amplified product [12,13]. In this sense, a duplex PCR, as performed in this study, avoids the variations in template starting amounts that can occur in independent PCR reactions, e.g., due to operator loading errors.

It is important to notice that this method requires that target and reference gene amplicons display a minimum difference between the melting temperatures, such that a clear individualization of the target and the reference gene melting peaks can be achieved. In our experience, this minimum difference was ≥5°C. Another important point to consider is that, similar to other amplicon-based gene dosage approaches, the duplex qPCR only assesses the copy number of the amplified region (90-220 bp), meaning that primers for each exon should be designed to screen the entire coding-sequence of a gene.

The novel assay presented here accurately detected losses and gains of one copy of the target sequence in all analyses (including mosaics of down to 25% of heterozygous mutated cells). Therefore, this methodology represents a reliable and flexible alternative both for screening gene dosage changes and as a validation assay for previously detected alterations. Nevertheless, validation of this technique for diagnostic purposes demands additional analytical studies in larger cohorts and for different genes.

## Conclusions

We have described a simple and inexpensive duplex qPCR method employing SYBR Green for the assessment of DNA copy number, thereby revealing genomic duplications and deletions. We also reported a FAP patient carrying a 5.2 MB deletion at 5q21.3-q22.3 in addition to a novel missense *APC* variant (p.Gln2322Arg). This gross deletion that encompasses the entire *APC* gene and 19 additional genes is likely to have been present in this family for at least three generations, with a quite unusual phenotype of absence of mental impairment and dysmorphic features.

## Competing interests

The authors GTT, FCCS, AVK, EMMS, BMR and BMC state that they have no competing financial interests.

## Authors’ contributions

Conceived and designed experiments: GTT, BMR, DMC; Performed and analyzed experiments: GTT, FCCS, ACVK, DMC; Ascertained and selected patients: EMMS, BMR; Contributed with reagents/materials/analyses tools: BMR, DMC; Wrote/edited the paper: GTT, FCCS, ACVK, DMC. All authors read and approved the final manuscript.

## Pre-publication history

The pre-publication history for this paper can be accessed here:

http://www.biomedcentral.com/1471-2350/13/55/prepub

## Supplementary Material

Additional file 1 **Methods, description of the Differential qPCR.** Figure S1. Click here for file
