# Supplementary material for: A novel SYBR-based duplex qPCR for the detection of gene dosage: detection of an APC large deletion in a familial adenomatous polyposis patient with an unusual phenotype
Source: BMC Med Genet. 2012 Jul 16;13:55. doi: 10.1186/1471-2350-13-55 (PMC3458979; doi:10.1186/1471-2350-13-55)
Supplement: Additional file 1 — Methods, description of the Differential qPCR. Figure S1. [file 1471-2350-13-55-S1.pdf]

Additional file 1

Methods:

Differential qPCR: 18 ng of each DNA sample was amplified in a 20 µL reaction containing 0.3 µmol of each primer and 1X SYBR® Green PCR MasterMix (Applied Biosystems). qPCR was performed on an ABI Prism 7500 detection system (Applied Biosystems) as following: preincubation of 95°C for 10 minutes and, 40 cycles of denaturation at 95°C for 15 seconds and annealing and extension at 60°C for 1 minute. Samples were amplified in duplicate for both target and normalizer genes. The average C_q of duplicates was used in the gene dosage ratio calculations. For each exon, four control individuals were used to calculate the average control C_q. Gene dosage ratios were calculated using the following equation: $2^{-[\Delta C\text{q (target)} - \Delta C\text{q (ref)}]}$, where ΔC_q (target) equals the difference between the C_q values for the patient and the control average for the target exon, and ΔC_q (ref) equals the difference between the C_q values for the patient and the control average for the reference gene. The $2^{-\Delta\Delta C\text{q}}$ method was performed for two *APC* exons (2 and 15) and for two reference genes: *GAPDH* intron 7 (12p13) and *HPRT1* exon 3 (Xq26.1). Ratios in the range 0.82 - 1.22 were considered normal, ratios between 0.41 and 0.61 indicated one copy deletion, and ratios $\geq$ 1.4 indicated amplifications (10% error in measured concentration).

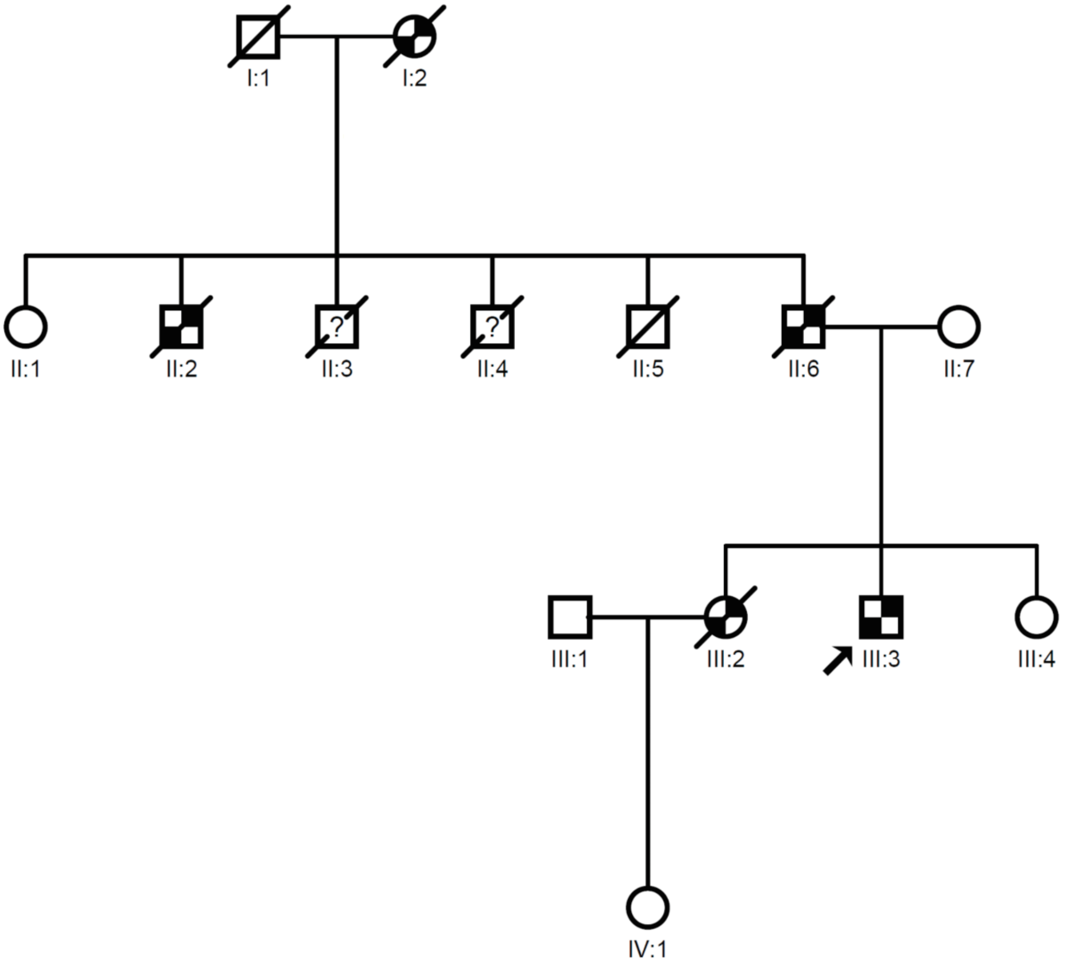

Supplementary Figure 1: Family pedigree of patient FAP02. The arrow indicates the index patient. All affected individuals presented both polyposis and colorectal cancer. The patient presented four affected deceased relatives: grandmother, uncle, father and one sister. One unaffected sister (III:4) and one unaffected niece (IV:1) were tested and neither the deletion nor the missense variant were detected. Genetic testing of the unaffected individual II:1 could not be performed. The symbol (?) indicates unknown cause of death in individuals II:3 and II:4.
